# The principal sources of William James' idea of habit

**DOI:** 10.3389/fnhum.2014.00274

**Published:** 2014-05-08

**Authors:** Carlos A. Blanco

**Affiliations:** Instituto de Cultura y Sociedad, Universidad de NavarraPamplona, Navarra, Spain

**Keywords:** James, habit, plasticity, empiricism

**A commentary on**

**The Principles of Psychology**

by James, W. (1890). New York, NY: Holt.

James consecrated the fourth chapter of his *Principles of Psychology* to the explanation of the idea of habit, for “when we look at living creatures from an outward point of view, one of the first things that strike us is that they are bundles of habits. In wild animals, the usual round of daily behavior seems a necessity implanted at birth; in animals domesticated, and especially in man, it seems, to a great extent, to be the result of education. The habits to which there is an innate tendency are called instincts; some of those due to education would by most persons be called acts of reason” (James, 1890).

The first relevant idea exposed by William James concerns the importance of plasticity in the development of all organic forms. Habit, enabled by this universally manifested–though in growing degrees- plasticity, is the biological correlate of the idea of natural law in the inanimate universe. In his own words, “the laws of Nature are nothing but the immutable habits which the different elementary sorts of matter follow in their actions and reactions upon each other.” Habit as the organic transposition of a natural law constitutes one of the guiding principles of James approach to this category. Its sources can be found in several authors. One of them is Léon Dumont (1837–1877), a French psychologist whose essay *De l'Habitude* (Dumont, 1876) is quoted by James in the above mentioned chapter. In this text, Dumont, following August Comte (1798–1857), had written that the idea of habit expresses, better than anyone else, the notion of a gradual acquisition of new faculties. According to him, the evolutionary perspective (recently discovered at his time) finds a good ally in the idea of habit, for it contains the progressive perfectibility of all beings, including man. In his studies of habit, sensibility and evolution, Dumont understood habit in analogy with the laws of inanimate nature.

A second major source of influence on James is the work of William Benjamin Carpenter (1813–1885), an English physician and physiologist who had done extensive work on comparative neurology. He spoke in terms of “adaptive unconscious” (Carpenter, 1874), in which there are resonances of Hermann von Helmholtz's (1821–1894) conception of thought and perception as drawing unconscious hypotheses and inferring probabilistic accounts about the surrounding environment (Helmholtz, 1867). According to this theory, thought and perception would operate, to a large extent, without awareness, and we would remain unconscious about a substantial body of mental phenomena which we consider rooted in the deepest powers of consciousness. As in the case of Dumont, in Carpenter there is a clear influence of Darwin's theory of evolution.

James conceived of a habit as the fruit of the exceptional plasticity of organic life, whose versatility would have played a significant role in favoring the adaption to different environment, needs, and challenges. But beyond the biological and evolutionary basis of habits, James wanted to unfold the formation of this kind of automatized behavior. To answer this question, he found inspiration in the work of English utilitarian philosophers like Alexander Bain (1818–1903) and John Stuart Mill (1806–1873). Bain, a Scottish psychologist and a leading figure of empiricism, had like Mill (whom he revered) endorsed an associationist approach to the acquisition of new behaviors (Bain, 1868).

James went a step further and delineated a refined view of habits in which the ideas of plasticity, automatization, and association were carefully bounded. For him, a habit corresponded to a general form of discharge that helped concentrate energies on unpredicted challenges. As he wrote, following Carpenter's idea that our nervous system grows to the modes in which it has been exercised, “habit simplifies the movements required to achieve a given result, makes them more accurate and diminishes fatigue” (James, 1890). In James' view, this is perhaps the most remarkable feature of a habit: it diminishes the conscious attention with which our acts are performed. The precedents to this idea can be found in the work of the French spiritualist philosopher François-Pierre Maine de Biran (1766–1824). According to James, the ability to act without the concourse of will has clear advantages. In a habitual action, mere sensation suffices for eliciting muscular movements, so that the upper regions of the brain and mind are set “comparatively free,” unless they go wrong and they immediately call our attention (James, 1890). This liberation shows extremely beneficial for displaying a larger array of actions.

## Conflict of interest statement

The author declares that the research was conducted in the absence of any commercial or financial relationships that could be construed as a potential conflict of interest.
